# In the Darkness

**Published:** 1872-05

**Authors:** 


### IN THE DARKNESS

Things are done which cause inscrutable diseases; and as every one who reads this article once will read it again, and many a dozen times, it is purposed to word it with great care, and make it plain enough to be understood fully by those whom it most concerns.

Whether in the palatial mansion of the city, or the hut of the forest, the little child is an angel of light and gladness; so ordained by Omnipotence, as the means of perpetuating the race, of peopling heaven with angels, gathered as redeemed ones from all of human kind that are born. There is an increasing number of husbands and wives who antagonize this design, on the ground that the expense, and trouble, and care of children make it necessary to limit the family.

All the means used impair the health of the wife; not a few endanger life. The wife suffers most, for she sins and is sinned against; not only is the body injured, but the moral sense is impaired.

The means oftenest employed are imperfect marital rights; cessation before the instant of completion, when the whole nature is aroused to the utmost tension, involving every fibre of the system. To arrest the progress instantaneously, throw back into the system and quench remorselessly what would have the next moment become a new life, and which in the lapse of time might have added glory to the race, is but a short remove from murder. The man must feel degraded in the act; the woman, in the aim to avoid the responsibilities for which she was created, must have remorse, at the moment the whole reproductive system is shocked in the disappointment of its appeasement.

In some of the Roman Catholic books of devotion there are many useful teachings in regard to such matters, and so delicately proposed, that only gross minds are excited wantonly. Two lessons are taught, which the whole Protestant world ought to know; lessons which, by being silently impressed on the mind by frequent reading and in devotional contemplation, come to have in time the preventive power of a direct revelation, attention to which is as beneficial to the physical nature as it is to the religious.

First, Whoever performs any act from the instant of conception, to destroy life, to blight and blast the flower, is guilty of

MURDER.

There is no prudish mincing of words; nothing is left indefinite, obscure; it is not a mere hint; it teaches that the destruction of a germ of immortality, whether it be a minute or a month old, whether before or after birth, is nothing less than

A GOD-PROVOKING MURDER.

This is the true doctrine, because the motive and the effect is the same; it is simply thwarting the purposes of the Almighty Father of us all, as plainly declared in his revealed Word, in the very beginning of human existence,— "Multiply and replenish." To destroy what would have been a life, is doing as much to thwart Omnipotence as to kill at any moment after life began; hence the far-reaching prescription of the second lesson inculcated; a lesson universally needed, a lesson universally disregarded, violated, every night of the world (see our book on "Sleep"). It was foreshadowed, at least it was in the mind's eye of the poet, when he wrote the lines,—

"Linked sweetness long drawn out."

The idea is, that the marital right, the act of reproduction, should be continuous until completed; no cessation for protracting gratification, because it baffles nature, it deranges the delicate machinery, and sometimes breaks it as effectually as a machine would be, which, revolving at the rate of a hundred revolutions in a second, is stopped still in an instant; it would break any mechanism to atoms. Nature cannot bear these violences with impunity, and hence are monstrosities of birth! The lesson is completed by announcing that the object should be, as much as possible, to comply with the requirement to "replenish," and as little as possible for the mere gratification of propensities.

This subject has been introduced thus into our pages with the utmost reluctance; but it has been so studiously avoided by the pulpit and the press, that a sentiment has actually grown up in the minds of many that if a child is destroyed any time before birth, there is no harm in it; that it is only murder when the full-born infant is strangled; while in reality, not only is that a murderous crime, but using means to prevent an immortal life, while indulging in the pleasures connected with it, is not less a murder, is not less a crime, a crime against the Infinite One, as its direct aim is to thwart his purposes.

